# Real-world outcomes of postmastectomy radiotherapy in breast cancer patients with 1–3 positive lymph nodes: a retrospective study

**DOI:** 10.1093/jrr/rrt084

**Published:** 2013-06-20

**Authors:** Imjai Chitapanarux, Ekkasit Tharavichitkul, Somvilai Jakrabhandu, Pitchayaponne Klunklin, Wimrak Onchan, Jirawattana Srikawin, Nantaka Pukanhaphan, Patrinee Traisathit, Roy Vongtama

**Affiliations:** 1 Division of Therapeutic Radiology and Oncology, Faculty of Medicine, Chiang Mai University, 110 Intawarorod Road, Chiang Mai, 50200, Thailand; 2 Department of Statistics, Faculty of Science, Chiang Mai University, 110 Intawarorod Road, Chiang Mai, 50200, Thailand; 3 St Teresa Comprehensive Cancer Center, Quail Lakes Dr, Stockton, CA 95207, California, USA

**Keywords:** postmastectomy radiotherapy, 1–3 positive nodes, breast cancer, Thai

## Abstract

Objective: To assess the treatment outcomes and to explore the determinants of clinical outcome in breast cancer patients with 1–3 positive nodes who did or did not receive postmastectomy radiotherapy (PMRT) in a tertiary care referral cancer center in Northern Thailand. Methods: We investigated a retrospective cohort of registered breast cancer patients at the Faculty of Medicine, Chiang Mai University, Thailand from 2001–2007. Analysis was performed using Cox regression models to identify factors affecting the overall survival (OS) and relapse-free survival (RFS) rates. Comparisons were made between two cohorts: women who received adjuvant PMRT (74 patients) and women who did not receive adjuvant PMRT (81 patients). Results: A total of 155 patients were included with a median follow-up period of 4.45 years. There was a statistically significant 4-year OS difference between the two groups of patients: 100% for the PMRT group and 93.1% for the non-PMRT group (*P* = 0.044). The 4-year RFS was 85.9% for patients receiving PMRT and 78.3% for patients who did not receive PMRT (*P* = 0.291). On multivariate analysis of OS, using hormonal treatment was the only significant independent factor associated with improved OS. On multivariate analysis of RFS, none of the variables were significantly associated with improved RFS. PMRT was notfound to be a prognostic variable related to the outcome of patients using a logistic regression model. Conclusion: Our retrospective, hospital-based analysis demonstrated that PMRT improved the treatment outcome in terms of OS for women with 1–3 node positive early-stage breast cancer.

## INTRODUCTION

Breast cancer is the most common cancer found in Thailand with 21 967 patients diagnosed in the years 2001–2003 [1]. In early breast cancer, the complete cure of disease and prevention of recurrence are the primary goals of therapy, and these can be achieved by a multimodality of approaches. Surgery is accepted as a standard treatment for patients with early-stage breast cancer and is usually followed by adjuvant chemotherapy or radiotherapy to control local recurrence arising from residual disease. Adjuvant chemotherapy has been proven to decrease the incidence of recurrence by 23% with a corresponding 15% decrease in mortality [2]. Adjuvant radiotherapy is another approach demonstrated in many randomized control studies to be effective in reducing local recurrence by 60–90% [3–4]. In early-stage breast cancer, the recurrence rate was reduced from 24% to 8.5% with the addition of radiation after breast-conserving surgery (BCS). The same benefit was also seen among those with high risk, as the local recurrence rate was reduced from 35% to 10% [5–7]. A study by the Early Breast Cancer Trialist's Collaborative Group (EBCTCG) in 2005 investigated the effects of adjuvant radiotherapy in early breast cancer patients. The 15-year mortality was seen to be significantly lower among patients receiving either breast-conserving therapy or mastectomy followed by irradiation [8]. Overgaard demonstrated that the benefit of postmastectomy irradiation (PMRT) was present regardless of the extent of disease [9]. In this study, PMRT resulted in a reduction in the 15-year locoregional recurrence rate from 51% to 10% among patients with ≥ 4 positive lymph nodes, and from 27% to 4% in patients with 1–3 positive lymph nodes, corresponding to a relative risk of 0.17 and 0.10, respectively [9]. However, immediate- and long-term adverse effects of PMRT are a major concern as significant numbers of deaths due to contralateral breast cancer, non-breast cancer, lung cancer and cardiac death have been observed among irradiated women [2, 8, 10]. Due to these concerns, debate on whether PMRT should be administered to those with < 4 nodes still exists. It remains controversial whether the risk of cardiac disease outweighs the disease-free survival benefit, buoyed by the inconsistency of results among different studies. Despite the controversy, clinicians for the most part have adopted the concept of adjuvant radiotherapy as a standard approach. However, there remains a paucity of evidence showing the real-world clinical outcomes of this practice in clinical settings. This retrospective cohort study was undertaken to assess clinical outcomes for those who have and those who have not received radiation therapy after surgery and/or chemotherapy/hormone therapy. The objectives of the study were to describe and to determine the differences in clinical outcomes in patients with 1–3 node positive early-stage breast cancer treated with and without PMRT.

## MATERIALS AND METHODS

The records of 1883 breast cancer patients who were treated at the Faculty of Medicine, Chiang Mai University between 2001 and 2007 were reviewed using a nonrandomized retrospective cohort study design. As this study's objective was to evaluate the real-world clinical outcomes of adjuvant radiotherapy in patients with early-stage breast cancer**,** we retrospectively collected such outcomes from the existing medical records. In our hospital, the indications for administering PMRT were locoregionally advanced stage at diagnosis, such as clinical stage T3–T4 and/or N2 or more, pathological ≥ 4 positive lymph nodes, and a close/positive surgical margin. The use of PMRT for 1–3 positive lymph nodes is dependent on a decision by the multidisciplinary team according to adverse pathological factors (e.g. < 50 years of age, negative hormonal status, or positive HER-2 status). The inclusion criteria for this study were patients who had: undergone modified radical mastectomy with or without adjuvant chemotherapy or adjuvant radiotherapy, Stage I–IIB breast cancer, pathological 1–3 axillary nodes positive, and negative resection margins. The exclusion criteria included: previous radiotherapy (breast or chest wall), incomplete treatment (either chemotherapy or radiotherapy), clinical N2 disease, pathologically revealed perinodal/extracapsular extension, metastatic breast cancer, or incomplete follow-up during the study period. The primary endpoints were 4-year relapse-free survival (RFS) and 4-year overall survival (OS). RFS was defined as the time from the date of primary surgery to the date of documented recurrence. OS was defined as the time from the date of primary surgery to the date of expiration. Locoregional recurrence was also assessed in the study group, although not as a primary endpoint. Locoregional recurrence was defined as recurrence at the skin or soft tissue over the ipsilateral chest wall or a recurrence at the ipsilateral regional lymphatic sites.

### Statistical analysis

Summary statistics for continuous variables were presented as categories. All outcomes for patients who had undergone PMRT were compared with those for patients who had not received PMRT. RFS and OS were estimated using the Kaplan–Meier method. The difference in 4-year RFS and 4-year OS between the two groups was determined using the Log-Rank test. In order to investigate the association between 4-year OS and baseline patient and treatment characteristics, univariate and multivariate Cox regression models were used. Using a stepwise backward approach to variable selection, we fit a Cox regression model to variables associated with the outcome (*P* < 0.20) in the univariate analysis. All *P*-values were two-sided, and *P*-values < 0.05 were considered to be statistically significant. All analyses were performed using STATA, version 10.1. (Stata Corp LP, Texas, USA).

## RESULTS

Among the 1883 primary breast cancer patients, 316 patients were identified as having 1–3 positive lymph nodes from surgical pathology specimen. Of those, 155 patients were eligible according to the inclusion and exclusion criteria (see above). Most of the patients (137/155, 88.4%) had received adjuvant chemotherapy. Among these, 63.2% had received a cyclophosphamide, methotrexate and fluorouracil (CMF) regimen, 35.5% had received an anthracycline regimen with or without a taxane. Adjuvant hormonal treatment was administered to 105 (67.7 %) patients. It was found that 74 (47.7%) patients had undergone PMRT while 81 (52.3%) patients had not received PMRT. The median number of axillary nodes removed was 13 and 15 in patients who had received PMRT and not received PMRT, respectively. The patient and treatment characteristics are shown in Table 1.

**Table 1.** Patient, treatment characteristics, and outcome of the treatment

Variables	No-adjuvant PMRT (81 patients)	Adjuvant PMRT (74 patients)
**Histological type**	0.283	
**Ductal**	76 (93.8)	68 (91.9)
**Lobular**	3 (3.7)	1 (1.4)
**Mucinous**	2 (2.5)	1 (1.4)
**Not assessed**	0 (0.0)	2 (2.7)
**Other**	0 (0.0)	2 (2.7)
**Histopathologic grade of tumor**	0.199	
**Well differentiated**	3 (3.7)	3 (4.1)
**Moderately differentiated**	52 (64.2)	39 (52.7)
**Poorly differentiated**	16 (19.8)	13 (17.6)
**Not assessed**	10 (12.3)	19 (25.7)
**Pathological tumor size**	0.132	
**Not available**	10 (12.4)	12 (16.2)
**< 1 cm**	0 (0.0)	2 (2.7)
**1–1.9 cm**	14 (17.3)	5 (6.8)
**2–3 cm**	39 (48.2)	33(44.6)
**>3 cm**	18 (22.2)	22 (29.7)
**Number of node examined**	0.898	
**1–5 nodes**	4 (4.9)	5 (6.8)
**6–10 nodes**	22 (27.2)	21 (28.4)
**11–15 nodes**	22 (27.2)	22 (29.7)
**> 15 nodes**	33 (40.7)	26 (35.1)
**Menopausal status**	0.966	
**Premenopausal**	31 (38.3)	30 (40.5)
**Postmenopausal**	45 (55.6)	39 (52.7)
**Uncertain**	5 (6.2)	5 (6.8)
**Estrogen receptor**	**1.000**	
**ER-negative**	**33 (40.7)**	**31 (41.9)**
**ER-positive**	**35 (43.2)**	**32 (43.2)**
**Not assessed**	**13 (16.1)**	**11 (14.9)**
**Progesterone receptor**	**0.550**	
**PgR-negative**	**27 (33.3)**	**31 (41.9)**
**PgR-positive**	**40 (49.4)**	**31 (41.9)**
**Not assessed**	**14 (17.3)**	**12 (16.2)**
**Chemotherapy**	**0.462**	
**No**	**11 (13.6)**	**7 (9.5)**
**Yes**	**70 (86.4)**	**67 (90.5)**
**Regimen**	**0.001**	
**AT based**	**19 (23.5)**	**36 (48.7)**
**Not AT based**	**62 (76.5)**	**36 (48.7)**
**No**	**0 (0.0)**	**2 (2.7)**
**Endocrine therapy**	**0.390**	
**No**	**29 (35.8)**	**21 (28.4)**
**Yes**	**52 (64.2)**	**53 (71.6)**
**Recurrence**	**0.138**	
**No**	**57 (70.4)**	**60 (81.1)**
**Yes**	**24 (29.6)**	**14 (18.9)**
**Site**	**0.882**	
**Distant metastases**	**15 (62.5)**	**10 (71.4)**
**Locoregional failure**	**5 (20.8)**	**3 (21.4)**
**Both**	**4 (16.7)**	**1 (7.1)**
**Status**	**0.126**	
**No evidence of disease**	**56 (69.1)**	**60 (81.1)**
**Death**	**6 (7.4)**	**1 (1.4)**
**Lost to follow-up**	**1 (1.2)**	**0 (0.0)**
**Living with disease**	**18 (22.2)**	**13 (17.6)**

**Fig. 1.**
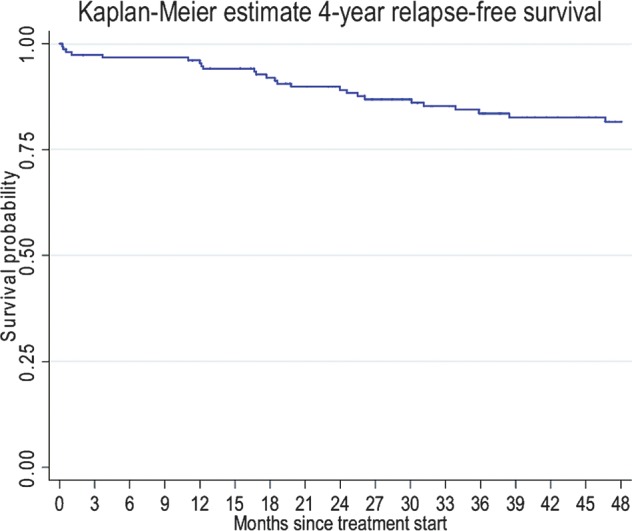
Four-year relapse-free survival rate.

**Fig. 2.**
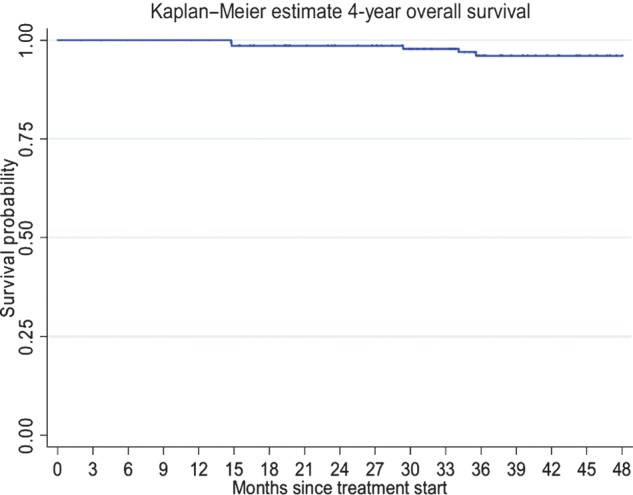
Four-year overall survival rate.

**Fig. 3.**
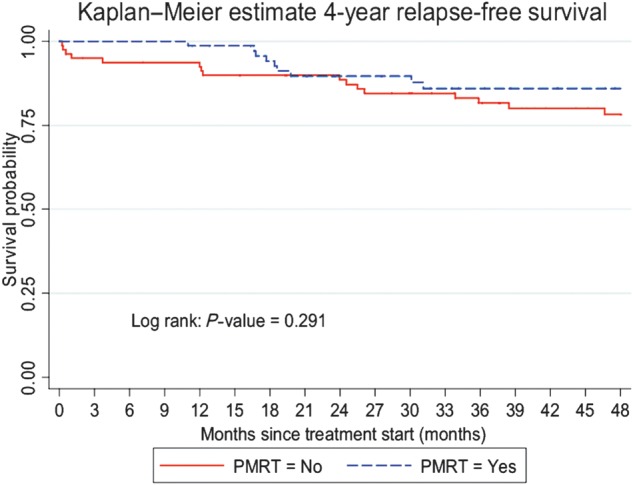
Four-year relapse-free survival rates by postmastectomy radiotherapy (PMRT).

**Fig. 4.**
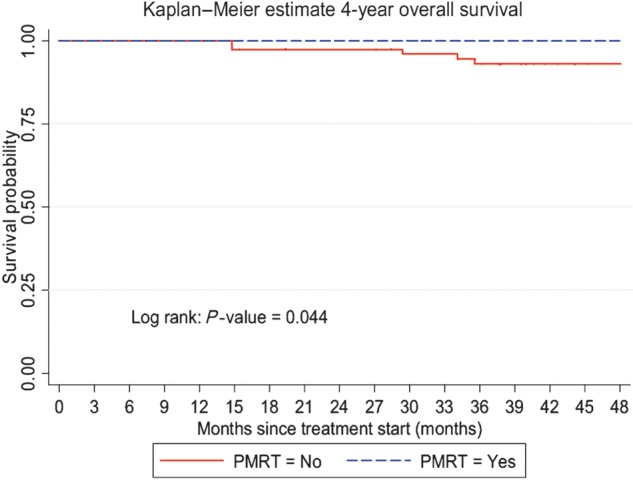
Four-year overall survival rates by postmastectomy radiotherapy (PMRT).

With the median follow-up of 4.45 years, 116 (74.8%) patients were alive without any evidence of disease, 31 (20.0%) patients were alive with disease, 7 (4.5%) patients were deceased and 1 (0.7%) patient was lost to follow-up. The 4-year RFS was 81.6% (95% CI, 73.8%–87.2%) (Fig. 1). The 4-year OS was 96.1% (95% CI, 90.8–98.4%) (Fig. 2). For patients who had and had not received PMRT, the locoregional recurrence rate was documented in 14 patients (18.9%) and 24 patients (29.6%), respectively. For patients who had received PMRT, the 4-year RFS was 85.9% (95% CI, 74.5–92.5%) and the 4-year OS was 100% (Figs 3 and 4). For patients who had not received PMRT, the 4-year RFS was 78.3% (95% CI, 66.9–86.2%) and the 4-year OS was 93.1% (95% CI, 84.1–97.1%) (Figs 3 and 4). The 4-year RFS rates were not significantly different between those who had received and had not received PMRT (85.9 vs 78.3%; *P =* 0.291). However, the 4-year OS rates were found to be significantly different (100 vs 93.1%; *P =* 0.044) between the two groups. On univariate analysis of RFS, having a positive progesterone receptor (PR) test, and receiving adjuvant endocrine therapy were significantly associated with improved RFS. The results of univariate analysis of RFS are summarized in Table 2. A multivariate analysis was performed to determine the contributing factors for RFS and no variable was found to be an independently significant factor for RFS (Table 2). Univariate analysis was then performed to identify factors affecting OS (Table 3). In brief, not using adjuvant hormonal treatment was a poor prognostic factor for OS. Multivariate analysis revealed that using hormonal treatment was the only significant independent factor for OS (Table 3). PMRT was not found to be prognostic with respect to RFS and OS using the logistic regression model.

**Table 2.** Univariate and multivariate analysis of the four-year relapse-free survival rate

Variables	Recurrent rate (n/N)	Univariate analysis			Multivariate analysis		
		Hazard Ratio: HR	95% CI for HR	*P*-Value	Hazard Ratio: HR	95% CI for HR	*P*-Value
Tumor Grade							
WD & MD	14.43 (14/97)	1	0.32–2.91	0.940			
PD	13.79 (4/29)	0.96					
Tumor size (cm)							
≤3	15.05 (14/93)	1	0.38–2.57	0.978			
>3	15.00 (6/40)	0.99					
No. of nodes							
≤15 nodes	17.71 (17/96)	1	0.33–1.78	0.539			
>15 nodes	13.56 (8/59)	0.78					
Menopausal status							
Premenopausal	14.75 (9/61)	1	0.56–2.92	0.564			
Postmenopausal	17.86 (15/84)	1.28					
ER status							
Negative	12.50 (8/64)	1	0.32–2.25	0.735			
Positive	11.94 (8/67)	0.84					
PgR status							
Negative	18.97 (11/58)	1	0.11–0.94	0.038	0.32	0.09–1.13	0.077
Positive	7.04 (5/71)	0.33					
Radiotherapy							
No	19.75 (16/81)	1	0.29–1.46	0.295			
Yes	12.16 (9/74)	0.65					
Chemotherapy							
No	16.67 (3/18)	1	0.29–3.27	0.972			
Yes	16.06 (22/137)	0.98					
Regimen							
A or A& T	10.91 (6/55)	1	0.70–4.39	0.230			
CMF	19.39 (19/98)	1.75					
Endocrine therapy							
No	26.00 (13/50)	1	0.15–0.74	0.006	0.82	0.25–2.70	0.740
Yes	11.43 (12/105)	0.33					

WD = well differentiated, MD = moderately differentiated, PD = poorly differentiated, ER = estrogen receptor, PgR = progesterone receptor, A = anthracycline, T = taxane, CMF = cyclophosphamide, methotrexate, 5-FU.

**Table 3.** Univariate and multivariate analysis of the four-year overall survival rate

Variables	% recurrence (n/N)	Univariate analysis			Multivariate analysis		
		Hazard Ratio: HR	95% CI for HR	*P*-Value	Hazard Ratio: HR	95% CI for HR	*P*-Value
**Tumor Grade**							
**WD & MD**	1.03 (1/97)	1	0.20–49.78	0.422			
**PD**	3.45 (1/29)	3.11					
**Tumor size (cm.)**							
**≤3**	1.08 (1/93)	1	0.40–49.26	0.222			
**>3**	5.00 (2/40)	4.46					
**No. of node examined**							
**≤15 nodes**	3.13 (3/96)	1	0.18–6.33	0.951			
**>15 nodes**	3.39 (2/59)	1.06					
**Menopausal status**							
**Premenopausal**	4.92 (3/61)	1	0.08–3.03	0.456			
**Postmenopausal**	2.38 (2/84)	0.51					
**ER status**							
**Negative**	3.13 (2/64)						
**Positive**	0.00 (0/67)						
**PgR status**							
**Negative**	1.72 (1/58)	1	0.05–12.35	0.855			
**Positive**	1.41 (1/71)	0.77					
**Radiotherapy**							
**No**	6.17 (5/81)						
**Yes**	0.00 (0/74)						
**Chemotherapy**							
**No**	11.11 (2/18)	1	0.04–1.26	0.089	0.25	0.04–1.53	0.135
**Yes**	2.19 (3/137)	0.21					
**Regimen**							
**A or A& T**	1.82 (1/55)	1	0.23–18.55	0.515			
**CMF**	4.08(4/98)	2.07					
**Endocrine therapy**							
**No**	8.00 (4/50)	1	0.01–0.87	0.037	0.11	0.01–0.96	0.046
**Yes**	0.95 (1/105)	0.10					

WD = well differentiated, MD = moderately differentiated, PD = poorly differentiated, ER = estrogen receptor, PgR = progesterone receptor, A = anthracycline, T = taxane, CMF = cyclophosphamide, methotrexate, 5-FU.

## DISCUSSION

It has been shown in multiple studies that radiotherapy reduces locoregional recurrence. The locoregional failure rate from the landmark trials ranged from 13–33% in this group of patients [11–14]. One of the possible reasons for the wide range in locoregional failure rate was the number of axillary nodes removed, which was low to moderate in some studies: a median of 7 in the Danish trial [9], and 11 in the British Columbia study [15], compared with higher numbers in the others. Our study had a relatively high number of nodes removed (a median of 13 nodes in patients who had received PMRT, and 15 nodes in patients who had not received PMRT).

Overgaard *et al*. reports a 114-month rate of locoregional recurrence of 26% for patients who did not receive radiotherapy and only 5% for those received radiotherapy [16]. The question is whether or not this locoregional recurrence decrease translates into a further benefit of OS. The benefit of radiotherapy on OS has been demonstratively shown in women of all ages with positive lymph nodes. However, it remains unclear whether this benefit is simply due to benefit in the ≥ 4 positive node group, in which there is already no controversy for the effectiveness of radiotherapy. This is an important question as a significant percentage of women in today's patient population present with 1–3 positive axillary nodes. They could be spared treatment sequelae (eg. lymphedema) if radiotherapy was found not to be effective.

In a Danish trial by Overgaard *et al*., in the 1–3 node positive patients, adjuvant RT improved locoregional control and also increased survival at 10 years by 17% [17]. At the median follow-up of 4.45 years, our retrospective study concurred with the Overgaard *et al.* result for OS, demonstrating a significant increase in OS with adjuvant radiotherapy compared with no radiotherapy (*P =* 0.044, Fig. 4). However, there was no significant difference seen for RFS (*P =* 0.291, Fig. 3).

In a retrospective study from Taiwan [18], Cheng *et al*. analyzed the incidence of locoregional failure in 125 postmastectomy patients with 1–3 positive axillary lymph nodes without adjuvant PMRT. Their 4-year locoregional recurrence rate was 16.1%, which was comparable to our result in the no-PMRT group (21.4%).

Cosar *et al*. [19] conducted a retrospective study of 90 patients with a similar design to and the same endpoints as our study, and demonstrated that PMRT in T1–2 and 1–3 axillary lymph node positive patients made a statistically significant improvement in RFS (*P =* 0.034), but no improvement in OS (*P =* 0.087).

Tendulkar *et al*. [20] reported the results from a retrospective review of 369 breast cancer patients with 1–3 positive lymph nodes, of whom 271 did not receive PMRT and 98 received PMRT. Their 5-year rate of locoregional recurrence (LRR) was only 8.9% without PMRT vs 0% with PMRT (*P* = 0.004). The difference in LRR seen between our study and that of Tendulkar *et al.* (29.6 and 18.9% vs 8.9 and 0%, respectively) can possibly be traced to the systemic therapy given. Tendulkar *et al.* reported that 70% of patients in their study received a modern systemic chemotherapy (Taxane), whereas in our study only 35% of patients received a Taxane-based chemotherapy regimen.

Most studies have concluded that locoregional treatment with PMRT improved survival by reducing locoregional failure rate [21–24]. Although we did find a statistically significant improvement in OS, our study found similar rates of locoregional failure between the two cohorts (20.8% in the PMRT group, and 21.4% in the no-PMRT group). This finding could be explained by the retrospective nature of our study, and also the smaller number of patients in our study than in others.

In our univariate analysis, not receiving chemotherapy and hormonal therapy were statistically significant factors associated with a lower OS rate. Lin *et al*. [25] reported tumor size, age and estrogen receptor (ER) status to be independent prognostic factors for OS in breast cancer patients with 1–3 axillary lymph node metastases in multivariate analysis. Adjuvant hormonal therapy turned out to be the only indicator with an independent impact on OS by multivariate analysis. Most likely due to the sample size limitation, our study could not demonstrate that PMRT was an independent prognostic factor for OS as determined by univariate analysis. For the 4-year RFS, although we did not find a statistically significant difference with and without PMRT, there was a trend to higher RFS in the PMRT group, especially for the first 20 months of the follow-up time (Fig. 3).

## CONCLUSION

In conclusion, our study reported a significant improvement in the 4-year OS rate with PMRT (100 vs 96.1%; *P =*0.04). It also showed a 7.6% improvement in the 4-year RFS rate with PMRT, although this result was not statistically significant. Our study is one of a number investigating treatment of breast cancer with 1–3 positive lymph nodes that supports the use of PMRT, especially in Asian women. The SUPREMO trial, a prospective evaluation of PMRT in this 1–3 node patient population subset, will hopefully provide clearer answers to this controversy.

## FUNDING

This study was supported in part by grant from F. Hoffmann-La Roche Ltd. (Thailand).
